# Pan-cancer analysis of ARID family members as novel biomarkers for immune checkpoint inhibitor therapy

**DOI:** 10.1080/15384047.2021.2011643

**Published:** 2022-03-03

**Authors:** Yan Zhu, Chun Yan, Xiaofei Wang, Zhijian Xu, Jianjian Lv, Xiaomei Xu, Wenjun Yu, Mi Zhou, Lu Yue

**Affiliations:** Department of Oncology, Qingdao Municipal Hospital, Qingdao, China

**Keywords:** ARID family, immune checkpoint inhibitors, overall survival, pan-cancer analysis, tumor mutation burden

## Abstract

Although immune checkpoint inhibitors (ICIs) have greatly improved cancer treatment, the accuracy of predictive biomarkers for ICI outcomes, such as PD-L1, TMB (tumor mutation burden) or MMR (mismatch repair) deficiency, have not been satisfactory. ARID family members are essential for maintaining the basic process of genomic stability and may serve as novel biomarkers for ICI therapy. A total of 1660 cancer patients who received ICI therapy were included in this pan-cancer analysis. The basic information and TMB values of each patient were collected. Survival analysis based on the Kaplan-Meier (KM) method was performed to explore the relationships between mutations in ARID family members and prognosis in pan-cancer as well as cancer subtypes. Genetic alterations in *ARID1A* (12%), *ARID1B* (5%), *ARID2* (6%) and *ARID5B* (2.6%) were identified in multiple cancer types. Patients harboring mutated ARID family members benefited more from ICI therapy (*P* = .0003). Mutated *ARID1A* (*P* = .01), *ARID1B* (*P* = .0097) and *ARID2* (*P = *.0054) all serve as compelling biomarkers in predicting the prognosis of ICI treatment. In addition, members of the ARID family were found to be strongly related to the abundance of CD4 + T cells and CD8 + T cells, the expression of PD-L1 and the TMB value in various cancers. Specifically, members of the ARID family could serve as novel biomarkers in multiple malignancies, especially gastrointestinal cancers. ARID family members serve as novel biomarkers for ICI therapy in malignancies. Testing the genomic status of ARID family members could help identify the definite subpopulation that benefits most from ICI treatment.

**Abbreviations:** AT-rich interactive domain (ARID)Switch/sucrose nonfermenting (SWI/SNF)Non-small cell lung cancer (NSCLC)Immune checkpoint inhibitors (ICIs)Tumor microenvironment (TME)Programmed death-ligand 1 (PD-L1)Tumor mutational burden (TMB)

## Introduction

Recent studies have suggested that immune checkpoint inhibitors (ICIs) significantly improve the prognosis of various types of malignancies with tolerable adverse events; thus, ICIs are now widely used clinically as standard therapies for malignancies.^1–3^ During large-scale clinical trials for ICIs, several biomarkers, such as the expression of programmed death-ligand 1 (PD-L1), the value of tumor mutational burden (TMB) and mismatch repair (MMR) deficiency, were found to be associated with sensitivity to ICI therapy.^4–6^ However, only a limited subpopulation of patients responded to ICI treatment, and the efficiency of these biomarkers in discriminating patients who would benefit was not satisfactory. Therefore, novel biomarkers for ICI therapy are warranted to enhance the response and improve the prognosis of a variety of malignancies.

The AT-rich interaction domain (ARID) family is a superfamily belonging to switch/sucrose nonfermenting (SWI/SNF) chromatin remodeling complexes and consists of a series of members associated with basic processes of cellular function, including the modification of chromatin structure and the regulation of targeted gene transcription.^7^ All ARID family members contain a DNA-binding domain through which they could bind targeted DNA and participate in the process of DNA replication, gene expression and cell growth, differentiation and development.^7–9^ According to our previous study,^10^ ARID1 subunits, especially mutated *ARID1B*, demonstrate compelling efficiency in predicting the prognosis of ICI therapy in advanced non-small cell lung cancer (NSCLC). Advanced NSCLC patients harboring mutated *ARID1* may develop a phenotype in the tumor microenvironment (TME) that is sensitive to ICI therapy, which is characterized by the enhancement of PD-L1 expression and elevation in the TMB score. Together, these features could identify patients likely to benefit from ICI therapies.

ICI therapy prolonged the overall survival (OS) of advanced NSCLC patients harboring mutated *ARID1*, but the role of ARID1 in other types of malignancies and other members of the ARID family in predicting the prognosis of ICI therapy remains to be elucidated. This pan-cancer study included a cohort of patients who received ICI therapy from a published study,^11^ and the results suggested that ARID family members are potential biomarkers that could potentially enhance the accuracy of prognosis and expand application of ICI therapy by using a pan-cancer analysis.

## Material and methods

### Study patients

A total of 1660 cancer patients who received ICI therapy were included in this pan-cancer study using data derived from the cBioPortal for Cancer Genomics.^11–13^ ICI therapies including atezolizumab, avelumab, durvalumab, ipilimumab, nivolumab, pembrolizumab or tremelimumab as monotherapy or in combination were administered to the patients in this study. In total, 99 patients received anti-CTLA-4 therapy, 1306 patients received anti-PD-1 or PD-L1 therapy, and 255 patients received a combination of anti-CTLA-4 and anti-PD-1/PD-L1 therapies. Specifically, 350 NSCLC patients, 320 melanoma patients, 151 renal cell carcinoma patients, 215 bladder cancer patients, 139 head and neck squamous cell cancer patients, 126 esophageal cancer patients, 117 glioma patients, 110 colorectal cancer patients, 44 breast cancer patients and 88 patients with unknown primary tumors were included. The basic information of the cohort of patients was collected. The OS data were measured from the date of the first administration of ICI therapy to the time of patient death or the most recent follow-up. The median follow-up time was 19 months, ranging from 0 to 80 months. All included patients gave their consent for the examination of targeted sequencing data.

### Bioinformatic and statistical analyses

TMB values were calculated with the total number of somatic nonsynonymous mutations, which was normalized to the total number of megabases sequenced according to a published study.^11^ The Kaplan-Meier (KM) method was used for survival analyses of the included patients, and the log-rank test was used to detect significant differences in survival. The exploration of the abundances of microenvironment components and the expression of PD-L1 was carried out with an online tool,^14^ and the Wilcoxon test was used to determine the differences. The genomic information was acquired from the cBioPortal for Cancer Genomics. All other statistical analyses were conducted by GraphPad Prism 8.0 software (GraphPad, La Jolla, CA), and Student’s t test was used to determine statistical significance. *P* values were determined by two-tailed tests. *P* < .05 was considered statistically significant.

## Results

### Pan-cancer analysis of the role of ARID family members in the prognosis of ICI therapy

**Figure 1.**
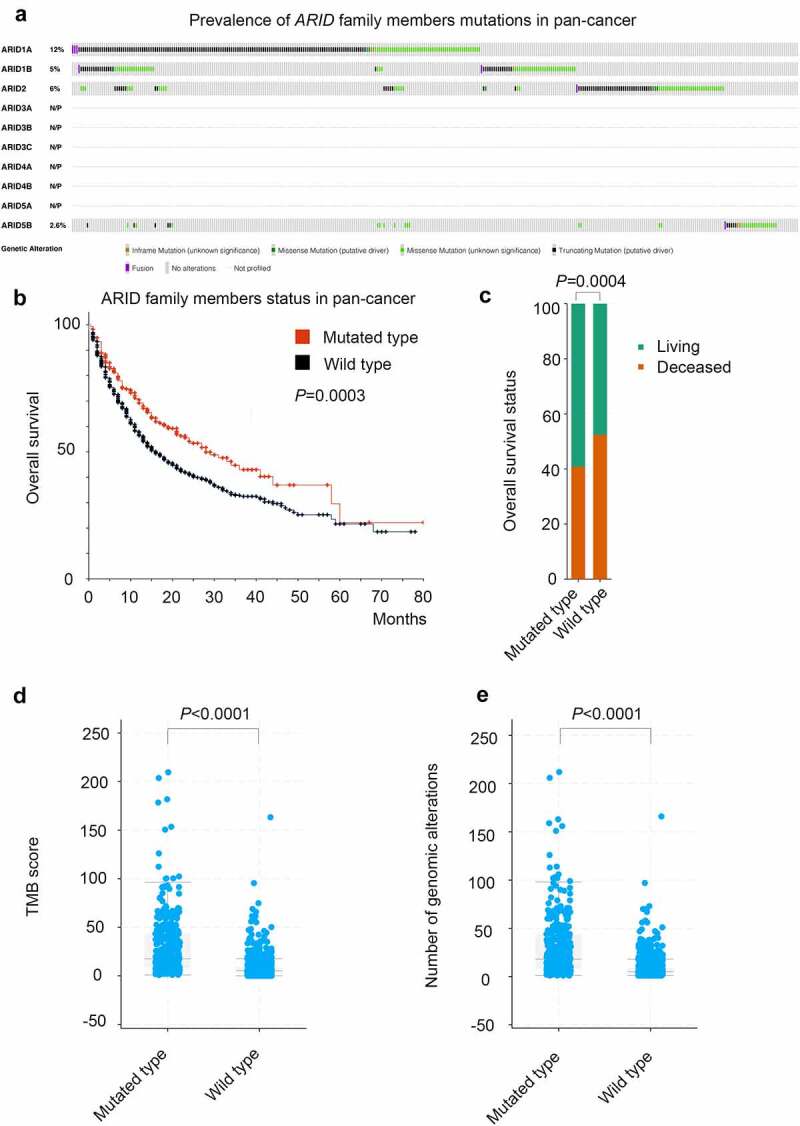
ARID family and its role in the pan-cancer analysis of cancer immunotherapy. a: The prevalence of genomic alterations in the ARID family; b: survival analysis based on genomic alterations in the ARID family in pan-cancer; c: the comparison of the overall survival status between the ARID mutated group and wild-type group; d: the comparison of tumor mutational burden (TMB) between the ARID mutated group and wild-type group; e: the comparison of mutation counts between the ARID mutated group and wild-type group.

**Figure 2.**
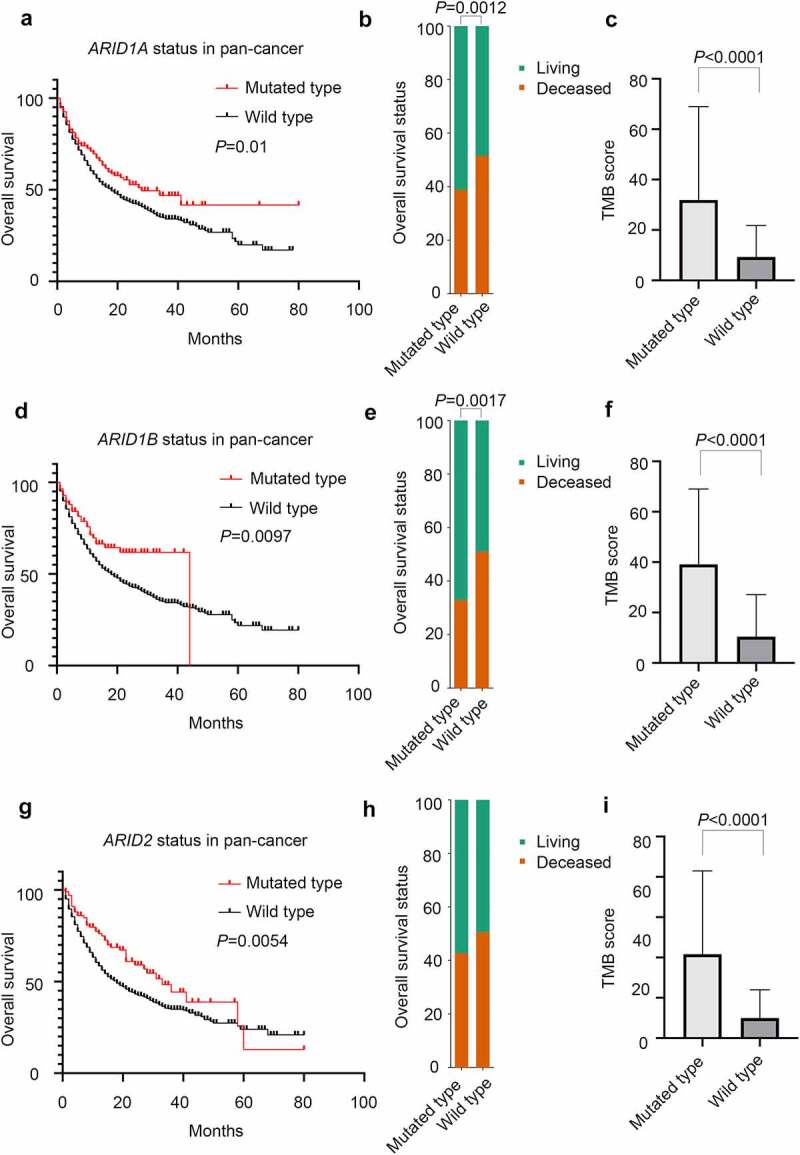
The role of ARID family subunits in the prognosis of ICI treatment in pan-cancer analysis. a: Comparison of the overall survival status between the ARID1A mutated group and wild-type group; b: survival analysis based on ARID1A mutation status; c: TMB score between the ARID1A mutated group and wild-type group; d: comparison of overall survival status between the ARID1B mutated group and wild-type group; e: TMB score between the ARID1B mutated group and wild-type group; g: comparison of the overall survival status between the ARID2 mutated group and wild-type group; h: survival analysis based on ARID2 mutation status; i: TMB score between the ARID2 mutated group and wild-type group.

Genomic alterations in ARID family members were not rare in this pan-cancer study. According to the targeted sequencing data of 1660 cancer patients, *ARID1A* (12%), *ARID1B* (5%), *ARID2* (6%) and *ARID5B* (2.6%) mutations are prevalent mutations in this family, as shown in Figure 1(a). Other members of the ARID family, such as *ARID3A/B/C, ARID4A/B* and *ARID5A*, were not mutated according to the data in this study. Divided by the *ARID* mutations, pan-cancer patients harboring mutations in ARID family members could benefit more from ICI therapy, and the median OS of these patients was significantly longer than that of patients with wild-type ARID family members (median OS: 28 months versus 16 months, *P* = .0003, Figure 1(b)). Specifically, patients harboring mutated *ARID1A* (median OS: 28 months versus 18 months, *P* = .01), mutated *ARID1B* (median OS: 48 months versus 18 months, *P* = .0097) or mutated *ARID2* (median OS: 33 months versus 18 months, *P* = .0054) benefited more from ICI therapy than patients with the wild-type counterparts, as shown in Figure 2(a,d,g), respectively. Detailed information for the pan-cancer survival analysis is presented in Table 1. The TMB value is likely a convincing biomarker in predicting the prognosis of ICI therapy, and patients with higher TMB values could benefit more from ICI therapy (median OS: 28 months versus 15 months, *P* < .0001). Other variables, such as ARID5B mutation status, age, sex and metastatic status of patients when the administration of ICIs was initiated, did not demonstrate satisfactory efficiency in predicting the prognosis of ICI therapy through pan-cancer analysis.

**Table 1.** Patients information in the pan-cancer analysis for cancer immunotherapy

Patients characteristics	Group	Patients number (%)	P value for OS comparison	P value for TMB comparison
***ARID1A* mutation**				
	mutated type	192 (12)	0.01	<0.0001
	wild type	1468 (88)		
***ARID1B* mutation**				
	mutated type	85 (5)	0.0097	<0.0001
	wild type	1575 (95)		
***ARID2* mutation**				
	mutated type	103 (6)	0.0054	<0.0001
	wild type	1557 (94)		
***ARID5B* mutation**				
	mutated type	44 (3)	0.1367	<0.0001
	wild type	1616 (97)		
**Age**				
	≥ 65	738 (44)	0.8444	0.0008
	< 65	922 (56)		
**Sex**				
	male	1033 (62)	0.1416	0.0603
	female	627 (38)		
**TMB score**				
	≥ 50th percentage	823 (50)	<0.0001	NA
	< 50th percentage	837 (50)		
**Metastasis**				
	metastasis	900 (54)	0.1713	0.1729
	no metastasis	760 (46)

Regarding the OS status (deceased or living) of pan-cancer patients shown in Figure 1(c), more patients in the mutated group of ARID family members than in the wild-type group survived at the last follow-up (*P* = .0004). For the individual members in this family, mutated *ARID1A* (*P* = .00124) and mutated *ARID1B* (*P* = .0017) demonstrated satisfactory efficiency in discriminating the outcome of patients, as displayed in Figure 2(b,e), respectively. As shown in Figure 2(h), mutated *ARID2* did not reach satisfactory efficiency in discriminating the outcome.

### Specific cancer analysis of the role ARID family members in the prognosis of ICI therapy

**Figure 3.**
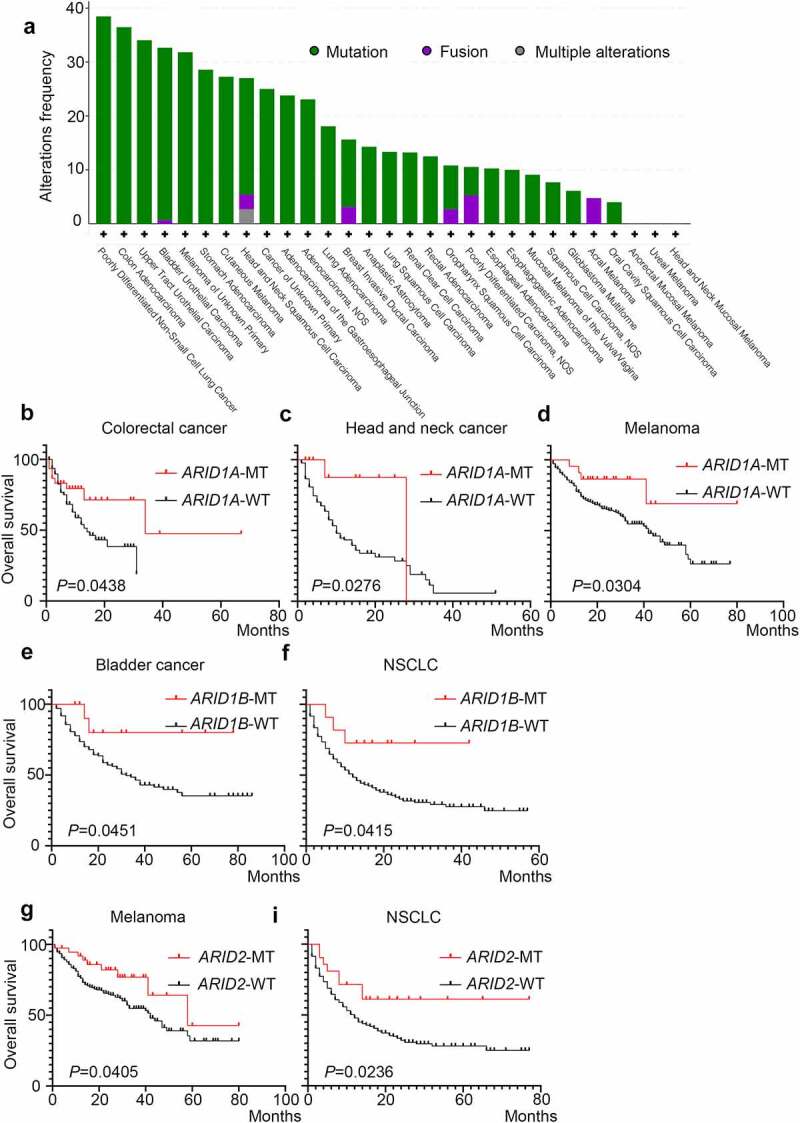
The role of ARID family subunits in the prognosis of immunotherapy in various cancer types. a: The prevalence of ARID family subunit mutations in various types of cancers; b-i: the role of ARID subunit mutations in the prognosis of immunotherapy in different types of cancers.

As shown in Figure 3(a), other than gene fusions, gene mutations in ARID family members, which serve as the main type of genomic alteration in this family, could be detected in a variety of malignancies, including NSCLC, colorectal cancer, melanoma, head and neck cancer and so on. This finding demonstrated that ARID family members could serve as advantageous biomarkers for ICI therapy in several malignancies according to the detailed analysis shown in Figure 3(b-i). Through the specific cancer analysis, colorectal cancer patients (*P* = .0438) and head and neck cancer patients (*P* = .0276) harboring *ARID1A* mutations had longer OS than wild-type patients, while both *ARID1A* mutations (*P* = .0304) and *ARID2* mutations (*P* = .0405) are suitable biomarkers for predicting the prognosis of melanoma patients. NSCLC patients harboring *ARID1B* mutations (*P* = .0415) or *ARID2* (*P* = .0236) mutations responded better to ICI therapy than wild-type patients. In addition, bladder cancer patients harboring *ARID1B* mutations (*P* = .0451) could achieve a better prognosis with ICI therapy.

### The role of ARID family members in the modulation of the tumor immune microenvironment

**Figure 4.**
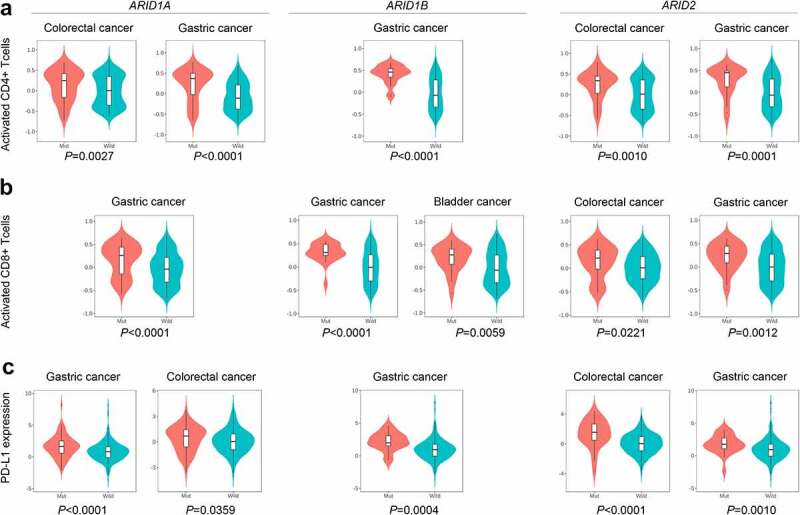
The role of ARID family subunits in the modulation of the tumor immune microenvironment. a:ARID mutated patients have increased CD4 + T lymphocytes in gastric cancer and colorectal cancer. b:ARID mutated patients have increased CD8 + T lymphocytes in various types of malignancies. c:Mutated *ARID1A*, mutated *ARID1B* and mutated *ARID2* were associated with the enhancement of PD-L1 expression in gastric cancer and colorectal cancer.

According to this pan-cancer analysis, ARID family members were found to be closely associated with the TMB score. Overall, patients harboring mutations in ARID family members had higher TMB values (*P* < .0001) and amounts of genomic alterations (*P* < .0001), as shown in Figure 1(d,e), respectively. Specifically, mutated *ARID1A* (mean TMB score.. 31.9 versus 9.3, *P* < .0001), mutated *ARID1B* (mean TMB score: 39.1 versus 10.5, *P* < .0001) and mutated *ARID2* (mean TMB score: 41.7 versus 10.0, *P* < .0001) in pan-cancer patients were all related to higher TMB values, as shown in Figure 2(c,f,i), respectively. In addition, pan-cancer patients harboring *ARID5B* mutations also had higher TMB values (mean TMB score: 47.3 versus 11.0, *P* < .0001). Additionally, elderly patients (>65 years) have higher TMB values (mean TMB score: 13.6 versus 10.6, *P* = .0008), as shown in Table 1. Further exploration, as shown in Figure 4, demonstrated the potential role of ARID family members in the modulation of cancer immunity. Mutated *ARID1A*, mutated *ARID1B* and mutated *ARID2* were all found to be associated with a higher abundance of CD4 + T lymphocytes and CD8 + T lymphocytes and the enhancement of PD-L1 expression in various types of malignancies, especially in gastrointestinal cancer. We further analyze the relationship between ARID1A/1B/2 mutations and DNA damage response genes, including BRCA1/2 and MMR related genes. ARID1A/1B/2 mutations were found to concur with DNA damage response genes as shown in **Figure S1** and **Table S1**.

## Discussion

Biomarkers for the prognosis of ICI therapy are a provocative topic in the era of cancer immunotherapy, and multiple biomarkers and mechanisms related to sensitivity to ICI therapy have been confirmed.^15^ With the development of targeted sequencing, it is known that the status of TMB and MMR, as well as the expression of PD-L1, serve as compelling biomarkers in ICI therapy.^4–6,16^ However, novel biomarkers still need to be further explored given the limitations of regular biomarkers.

Previous studies have focused more on the essential role of ARID1A in guiding the treatment of ICIs. Through the NGS examination reported by the MYSTIC trial,^17^ *ARID1A* mutations are associated with improved outcomes with the PD-1 inhibitor durvalumab plus the CTLA4 inhibitor tremelimumab. In addition, correlations with mutated ARIDA might improve prognostic interpretation in patients receiving ICI therapy for multiple types of malignancies.^18,19^ However, whether other members of the ARID family have the same effect in predicting the prognosis of ICI therapy remains to be further studied. Through this pan-cancer analysis, the important role of ARID family members in cancer immunotherapy was explored. Multiple members in this family demonstrated their ability to predict the prognosis of ICI treatment. Generally, mutated *ARID1A, ARID1B* and *ARID2* were all related to the good prognosis of ICI therapy based on the pan-cancer population. This finding suggests that mutations in ARID family members serve as protective factors for cancer immunotherapy. In addition, the prognostic value of ARID family members in different types of malignancies varies. Malignancies derived from the lung, gastrointestinal tract (GIT), urogenital tract (UGT) and melanocytes more easily harbor genomic alterations in ARID family members. Meanwhile, these findings suggested that better prognostic efficiency could be acheived by evaluating ARID family members in these types of malignancies and thus improve prognosis and expand the benefit population.

The tumor immune microenvironment has been suggested to be closely associated with the biological behaviors of malignancies^20^ and participates in the phenotype classification of cancer immunotherapy.^21^ Tumor-infiltrating lymphocytes (TILs) and the expression of PD-L1 are critical factors for discriminating the different subtypes of the TIME and the response to ICI treatment. Previous studies suggested that *ARID1A* mutations are likely to be related to the higher immune infiltrates in endometrial cancer, stomach cancer and colon cancer.^22^ In addition, in our previous study,^10^ we noticed that the expression of ARID1A and ARID1B is closely associated with the abundance of TILs, including CD4 + T cells and CD8 + T cells. Therefore, we further explored this finding in this pan-cancer analysis and investigated the relationship between mutations in ARID family members and the abundance of TILs. According to the results of the pan-cancer analysis, we found that the abundances of CD4 + T cells and CD8 + T cells were significantly elevated in the mutated *ARID1A*, mutated *ARID1B* and mutated *ARID2* groups, especially for colorectal cancer and gastric cancer. In addition, the enhanced PD-L1 expression of patients harboring mutations in *ARID1A, ARID1B* or *ARID2* could be detected. This suggests that members of the ARID family play important roles in the modulation of the TIME and that mutations in these genes might influence the outcome of ICI therapy by changing the balance of the TIME into the ICI-sensitive phenotype. Besides, ARID1A/1B/2 mutations were found to concur with DNA damage response genes. This might disclose the potential mechanism which ARID1A/1B/2 mutations were associated with High TMB.

## Conclusions

In conclusion, ARID family members might serve as novel biomarkers for ICI therapy in malignancies. Cancer patients harboring mutated *ARID1A, ARID1B* or *ARID2* benefit more from cancer immunotherapy. Mutated members of the ARID family play important roles in the modulation of cancer immunity and might influence the phenotype of the TIME.

## Data Availability

All data generated during this study are included in this published article. The datasets supporting the conclusions of this article are available in the cBioportal for Cancer Genomics.
